# An efficient and accurate multi-level cascaded recurrent network for stereo matching

**DOI:** 10.1038/s41598-024-57321-6

**Published:** 2024-04-08

**Authors:** Ziyu Zhong, Xiuze Yang, Xiubian Pan, Wei Guan, Ke Liang, Jing Li, Xiaolan Liao, Shuo Wang

**Affiliations:** School of Mechanical Engineering, Guangxi University, Nanning, 530004 Guangxi China

**Keywords:** Disparity estimation, Stereo matching, Transformer, Convolutional neural network, Computer science, Information technology

## Abstract

With the advent of Transformer-based convolutional neural networks, stereo matching algorithms have achieved state-of-the-art accuracy in disparity estimation. Nevertheless, this method requires much model inference time, which is the main reason limiting its application in many vision tasks and robots. Facing the trade-off problem between accuracy and efficiency, this paper proposes an efficient and accurate multi-level cascaded recurrent network, LMCR-Stereo. To recover the detailed information of stereo images more accurately, we first design a multi-level network to update the difference values in a coarse-to-fine recurrent iterative manner. Then, we propose a new pair of slow-fast multi-stage superposition inference structures to accommodate the differences between different scene data. Besides, to ensure better disparity estimation accuracy with faster model inference speed, we introduce a pair of adaptive and lightweight group correlation layers to reduce the impact of erroneous rectification and significantly improve model inference speed. The experimental results show that the proposed approach achieves a competitive disparity estimation accuracy with a faster model inference speed than the current state-of-the-art methods. Notably, the model inference speed of the proposed approach is improved by 46.0% and 50.4% in the SceneFlow test set and Middlebury benchmark, respectively.

## Introduction

Estimating depth from rectified stereo image pairs is a key technology for many fields, such as robot navigation, autonomous driving, augmented reality, and 3D reconstruction^1–3^. The key to depth estimation is stereo matching, i.e. first calculating the disparity between the pixels of a pair of rectified stereo images and then finding the depth of that pixel by triangulation^4–6^.

Traditional stereo matching is concerned with designing better matching costs and corresponding efficient inference algorithms, mainly divided into global stereo matching methods^7,8^ and local stereo matching methods^2,9^. In general, the global approach^8^ has a higher accuracy of disparity estimation than the local approach^9–11^, but it comes with higher computational complexity. Hirschmuller proposed a Semi-Global Matching (SGM) method over mutual information^12^, reducing computational complexity and maintaining higher accuracy simultaneously. However, in complex scenes with areas such as texture-free regions, thin structures, and repetitive features, traditional stereo matching approaches have much lower accuracy than learning-based methods^1,13^. Recently, learning-based stereo matching methods have made breakthroughs in terms of disparity estimation accuracy^14–17^, and the Transformer-based cascaded recurrent network has taken the disparity estimation accuracy to a new height^16^. However, with the high computational cost, it is difficult to utilize in practice. This method still faces enormous challenges in practical scenarios requiring high accuracy and efficiency, such as robot navigation and autonomous driving.

In the process of performing model inference, achieving high accuracy disparity estimation of stereo image pairs is extremely difficult: (1) The captured stereo image pairs are difficult to ideally rectify^18^ because the camera module will have problems with focal length and distortion parameters and inconsistencies on the left and right cameras, which will inevitably result in erroneous calibration. (2) Accurate recovery of texture-less regions, thin structures, and repetitive features is a highly complex problem^1^, especially for high-resolution stereo image pairs, where erroneous features around image details and detail degradation due to up-sampling and down-sampling further increase the difficulty of disparity estimation^16,19^. Additionally, achieving faster model inference while ensuring better disparity accuracy is more challenging. Existing methods^6,16,20^ add relevant model components to achieve high accuracy, which dramatically increases the complexity and computation of the network and makes it harder to achieve faster model inference.

Based on the above problems, we propose LMCR-Stereo, namely a Lightweight-based multi-level Cascaded Recurrent (LMCR) Stereo matching network, which includes efficient modeling and multi-level network refinement design to solve the problem of fast and accurate balance of stereo matching. The overall design is based on CREStereo^16^. To better recover the detailed features of complex images, we design a multi-level network with hierarchical recurrent refinement and cascaded refinement in a coarse-to-fine manner and then continuously update the disparity estimation by recurrent refinement. Meanwhile, to make the training process of the model achieve convergence quicker and have a higher inference speed, the disparity values are first initialized, followed by extracting a three-layer feature pyramid with image resolutions of 1/32, 1/16, and 1/8. Finally, two strategies, the adaptive group correlation layer and lightweight group correlation layer, are used to update the disparity estimation at different resolutions. Besides, we design a pair of Slow-Fast multi-layer cascaded stacked inference structures for disparity prediction with the most suitable model input size and corresponding inference strategies to make our method have better generalization ability and faster model inference speed.

The summary of our main contributions is as follows: (1) Designed an efficient and accurate multi-level cascaded recurrent network applied to stereo matching. (2) Introduced a pair of efficient group correlation layer modules to speed up the model prediction time significantly. (3) Proposed a slow-fast multi-level cascaded recurrent stacked inference structure to make the model generalize better. (4) Compared with the original CREStereo^16^ method, our method improves the accuracy and speed of the SceneFlow dataset by 19.6% and 46.0%, respectively. It improves the model inference speed by 50.4% on the Middlebury benchmark test.

## Results

There are some metrics for model evaluation. The same metrics may have different names in different datasets, and we use the same name for all throughout this paper. Bad pixel percentage (Bad) represents the percentage of pixels with disparity error larger than a certain threshold. There are multiple thresholds for this metric. For example, Bad 1.0 considers all pixels with errors greater than 1 pixel, and Bad 2.0 considers errors greater than 2 pixels, etc. In the Middlebury benchmark, Bad 2.0 is the default metric being used for overall ranking. Average absolute error in pixels (AvgErr) calculates the average disparity error for all bad pixels. Root-mean-square disparity error in pixels (RMS) measures the square root of the average of the squared disparity errors. Error quantile in pixels shows the distribution of disparity errors and different error quantiles include A50, A90, A95, and A99. The A99 quantile represents the value below which 99 percent of the disparity errors fall. Middlebury provides the total runtime (Time) for each method, and also the Time per Megapixel (Time/MP) and Time per Gradient Descent Iteration (Time/GD). Time represents the total time required by the algorithm to process the entire image, which provides an overview of the algorithm’s efficiency. Time/MP helps to analyze the algorithm’s efficiency in a size-independent manner. Time/GD indicates the efficiency of the optimization process, which is significant to the iterative refinement scenarios. In this paper, we focus on Time and Time/GD. In SceneFlow dataset, number of parameters (M) is given to show the model complexity, capacity to learn, and potential computational requirements.

### Middlebury benchmark

**Figure 1:**
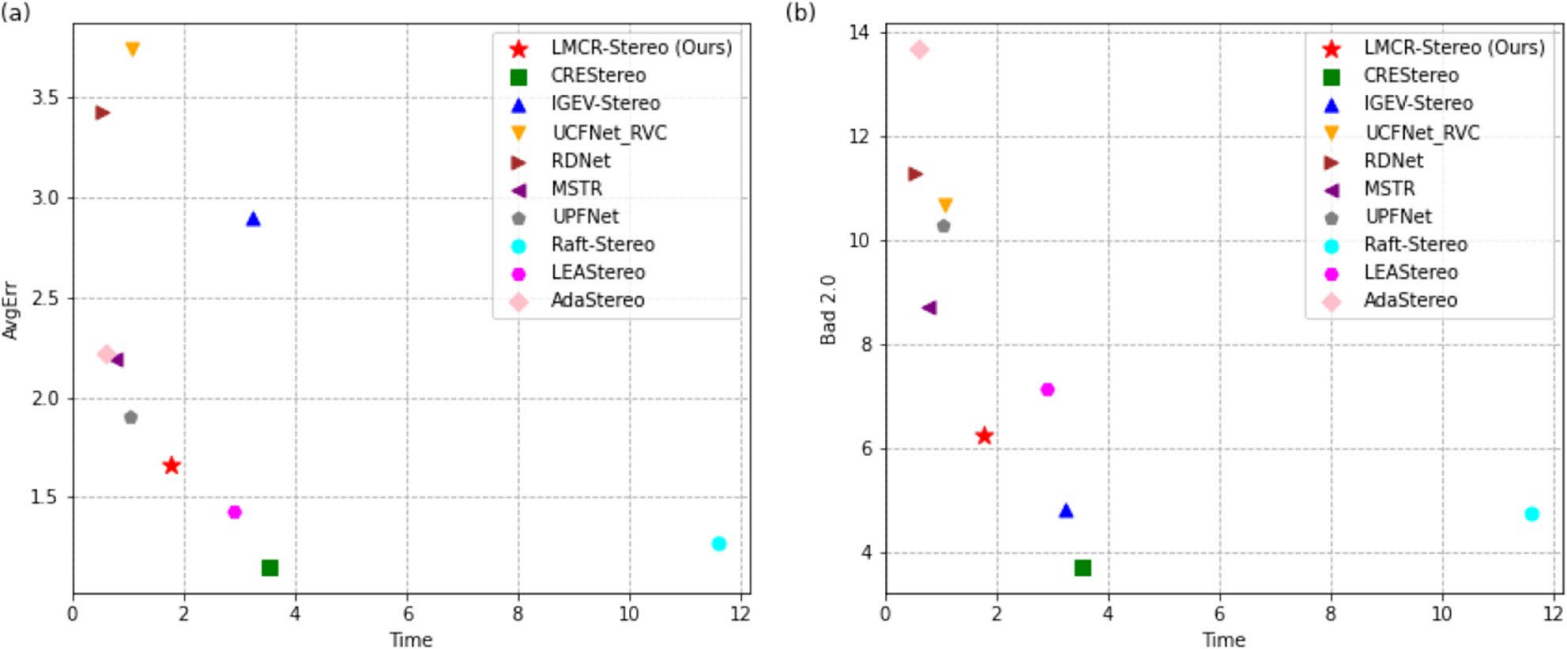
The scatter plot (**a**) comparing average end-point error vs. total inference time, and (**b**) Bad 2.0 vs total inference time on Middlebury benchmark. The figure shows that our proposed approach achieves a better balance between accuracy and speed.

So far, the model inference time of LMCR-Stereo is reduced by 50.4% compared to the CREStereo^16^ approach. As shown in Fig. 1, we evaluate existing advanced stereo matching algorithms and proposed LMCR-Stereo method, including AvgErr versus model inference time, and Bad 2.0 versus model inference time. Our proposed method achieved a better balance between model prediction speed and disparity estimation accuracy.

**Figure 2:**
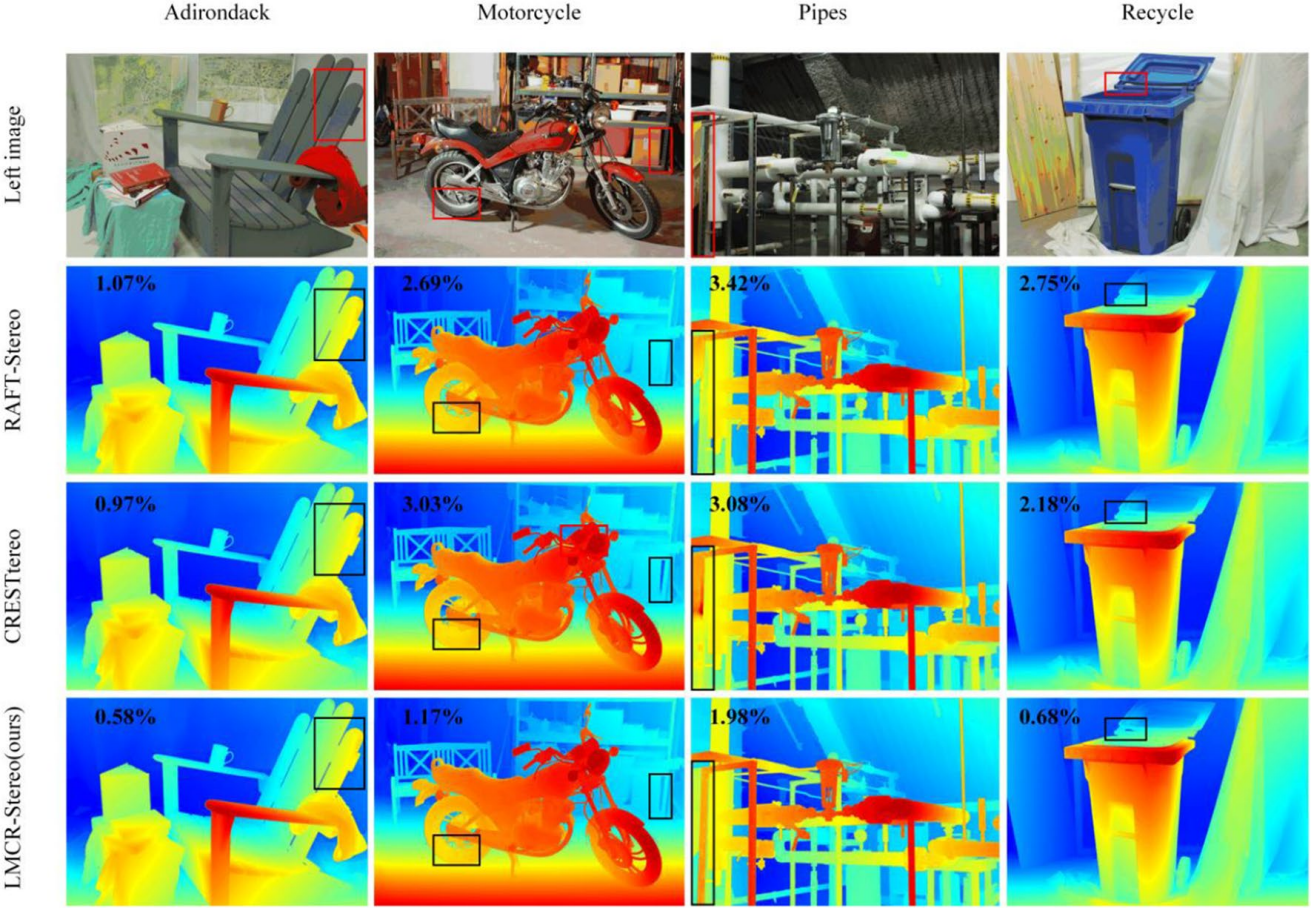
Comparison of results from different methods on Middlebury training set. From top to bottom: left images of stereo pair, results of RAFT-Stereo, results of CREStereo, and results of our method. In the results of each method, the number in the upper left corner of the picture represents the Bad 2.0 metric. The red box in the left figure is the same area as the box in the results.

We pre-increased 23 pairs of images from the Middlebury 2014 dataset (including 13 additional pairs of ground truth images) to the same number as the simple CREStereo training set using a data enhancement method. Our network was then trained using both the augmented dataset and the simple CREStereo training set. We use the pre-trained model for 300,000 training iterations, predict the test set and the training set using a multiple of 64 with full-resolution image resizing, and evaluate the training set and the test set using resized full-resolution images. The training set is estimated using a two-stage slow version of the inference structure, and the test set uses a two-stage fast version. We submit the predictions of our trained model to the online leaderboard. Compared with more than 120 other methods, we achieve state-of-the-art performance in most metrics in the training set. In the test set, although our proposed method does not surpass the prediction accuracy of the current state-of-the-art methods, the Time/GD metric is improved by 58.2% compared to the performance of CREStereo, which dramatically improves the speed of model prediction and ranks high in almost all metrics. The quantitative comparison results are shown in Tables 1 and 2. Figure 2 compares our proposed method in the training set with other methods. Our approach is more accurate in the contour detail part of the training set images and the overall disparity prediction.

**Table 1:** Quantitative results on Middlebury benchmark.

Method	Bad 0.5	Bad 1.0	Bad 2.0	Bad 4.0	AvgErr	RMS	A99	Time	Time/GD
LMCR-Stereo (Ours)	36.4	13.20	6.27	3.72	1.66	8.98	31.3	1.76	0.93
CREStereo^16^	28.0	8.25	3.71	2.04	1.15	7.70	22.9	3.55	2.22
IGEV-Stereo^21^	32.4	9.41	4.83	3.33	2.89	12.80	43.0	3.23	1.64
UCFNet_RVC^22^	51.8	25.70	10.70	6.13	3.74	16.50	88.6	1.08	2.20
RDNet^23^	53.6	26.50	11.30	5.77	3.42	15.70	82.2	0.55	1.37
MSTR^24^	48.5	21.60	8.72	3.99	2.19	13.80	45.7	0.76	1.52
UPFNet^25^	52.5	25.70	10.30	4.58	1.90	10.20	32.4	1.04	2.09
RAFT-Stereo^20^	27.7	9.37	4.74	2.75	1.27	8.41	21.7	11.60	5.76
LEAStereo^26^	48.2	20.80	7.15	2.75	1.43	8.11	20.2	2.90	7.27
AdaStereo^27^	65.5	29.50	13.70	6.35	2.22	10.20	40.6	0.60	0.38

**Table 2:** Quantitative results on Middlebury training set.

Method	Bad 0.5	Bad 1.0	Bad 2.0	Bad 4.0	AvgErr	RMS	A99	Time	Time/GD
LMCR-Stereo (Ours)	25.1	8.05	3.12	1.50	0.64	3.14	7.49	1.66	1.18
CREStereo^16^	26.2	8.73	4.01	2.04	0.94	5.21	17.10	3.66	2.98
IGEV-Stereo^21^	20.3	6.85	3.61	2.37	1.44	5.24	14.10	3.06	2.11
UCFNet_RVC^22^	43.9	17.90	8.31	4.56	2.22	8.31	33.60	1.01	2.75
RDNet^23^	43.6	17.90	6.87	3.00	1.60	6.18	20.40	0.55	1.80
MSTR^24^	32.6	11.80	4.43	1.90	1.37	9.91	25.70	0.75	1.97
UPFNet^25^	40.1	15.50	5.64	2.10	1.05	5.39	13.20	1.00	2.74
RAFT-Stereo^20^	28.6	10.60	5.25	2.89	1.04	5.25	19.10	11.00	7.37
LEAStereo^26^	44.6	18.40	6.94	2.62	1.09	4.99	13.30	2.90	9.48
AdaStereo^27^	58.4	31.30	14.30	5.82	1.92	7.66	31.70	0.60	0.49

### SceneFlow dataset

**Figure 3:**
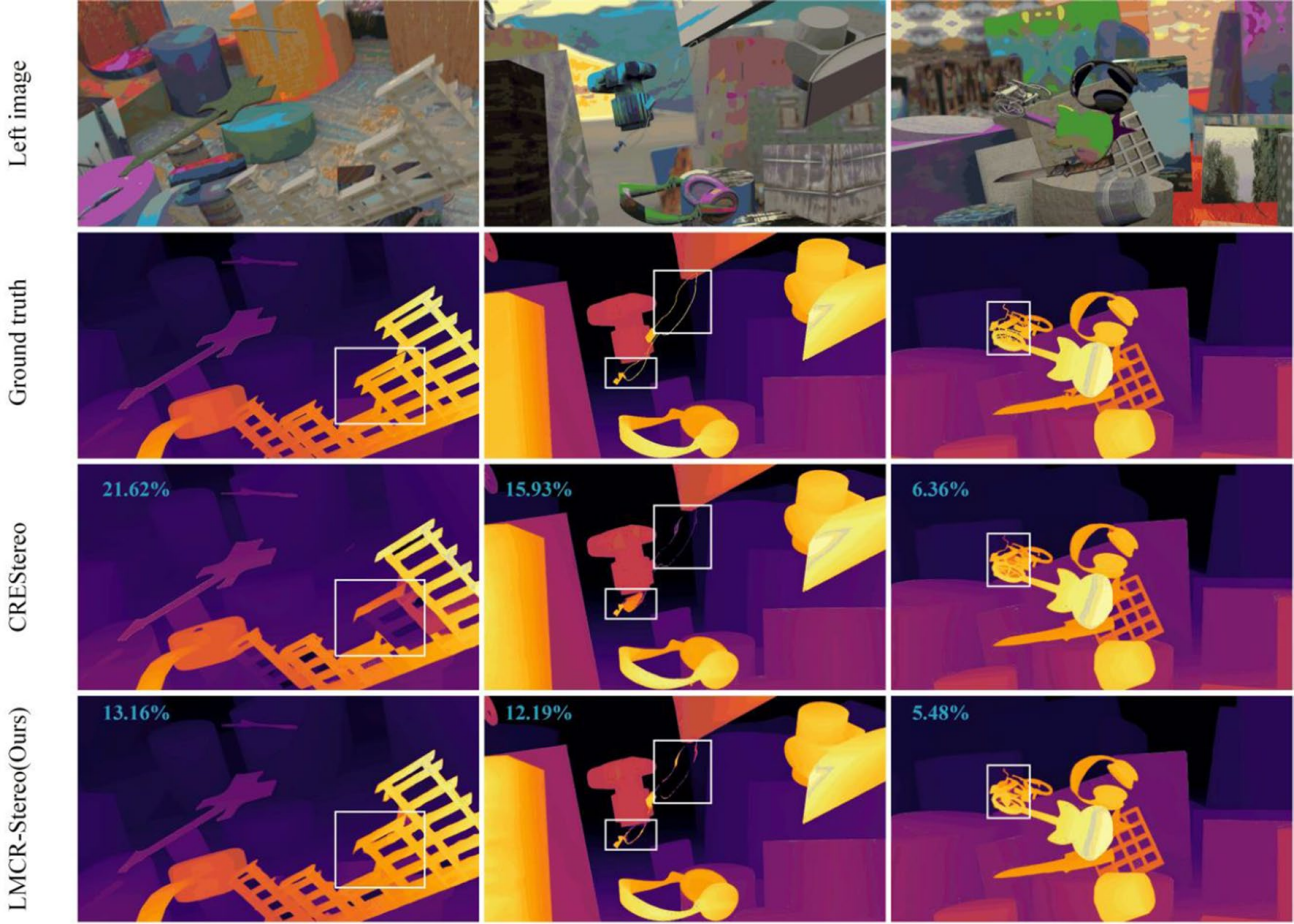
Comparison of results from different methods on SceneFlow test set. From top to bottom: left images of stereo pair, ground truth disparity maps, results of CREStereo, and results of ours. In the results of each method, the number in the upper left corner of the picture represents the Bad 1.0 metric.

Using SceneFlow, a large synthetic dataset, we train the LMCR-Stereo network with both “finalpass” and “cleanpass” versions and test the disparity estimation effect after training with “finalpass”, which contains 35,454$\times$2 training image pairs and 4,370 test image pairs. We set [$n_{1}$, $n_{2}$, $n_{3}$, $n_{4}$] to Type 6 for the training process with 300,000 iterations and use the trained model as a pre-trained model. We use a model input size of 768$\times$1024 and single-stage inference to predict the test set disparity values and set $n_{3}$ to 2. For a fair comparison, we also trained CREStereo^16^ using the same environment. As shown in Table 3, our proposed approach achieves disparity estimation accuracy beyond that of CREStereo^16^, which improves by 13.3% and 19.6% at AvgErr and Bad 1.0 metrics, respectively. In addition, the model inference speed increases by 46.0%. As shown in Fig. 3, our proposed method achieves good prediction results on contour edges, fine features, and higher overall accuracy estimates.

**Table 3:** Quantitative results on SceneFlow test set.

Method	AvgErr	Bad 1.0	Bad 3.0	Bad 5.0	Parameters (M)	Time (s)
LMCR-Stereo (Ous)	0.867	8.15	3.71	2.58	6.69	0.450
CREStereo^16^	0.999	10.14	4.36	3.00	5.43	0.833

## Discussion

This paper proposes a fast and accurate multi-level cascaded recurrent stereo matching network. The unique multi-level recurrent cascaded iterative architecture obtains more accurate disparity prediction values by exploiting the interaction of high and low-resolution features with the same number of parameters. In addition, based on the idea of efficiency, we propose a pair of group correlation layers, which can significantly reduce the computation of the model. We also design a slow-fast multi-level cascaded stacked inference structure, which can select the most suitable inference structure according to the different scenario data. Under the same experimental conditions, the disparity estimation accuracy and model inference speed are improved by 19.6% and 46.0%, respectively, in the SceneFlow test set. Although the accuracy on the Middlebury benchmark still needs to be improved, the model inference speed is enhanced by 50.4%. However, our model inference still struggles to reach real-time inference on high-resolution stereo image pairs, and our method may have an over-fitting issue in Middlebury training set, which requires adjustment on training parameters. In addition, our method was trained via a cloud computing service provider, and the service provider may not have given us full computer resources, which may have resulted in errors in operating speed. For future works, we will aim to pursue more efficient and accurate stereo matching networks in the future.

## Related works

In learning-based stereo matching tasks, achieving better accuracy and faster speed usually involves two implications: (1) How to design a more accurate stereo matching network to obtain a more precise disparity estimation. (2) How to design an efficient model component and a fast inference structure so that the model can perform disparity estimation faster. Designing high-precision network models^16,20^ and efficient model components^20,28,29^ are the exploratory directions for many current models.

Since the first introduction of convolutional neural networks (CNNs) to stereo matching tasks by Žbontar and LeCun^30^, researchers have been dedicated to exploring network models with higher disparity estimation accuracy. Inspired by traditional methods, many researchers^6,19,31–33^ have used 3D convolutional network architectures for end-to-end stereo matching, such as PSMNet^6^, GANet^19^ and ACFNet^31^, have achieved state-of-the-art performance. Based on GCNet^32^, Chang et al. proposed a stacked hourglass module and a pyramidal feature extraction network^6^. This network improves the disparity estimation accuracy of the model. Where GCNet constructed a four-dimensional cost volume using $height\times width\times (\max disparity + 1 )\times feature size$. Based on PSMNet^6^, GANet^19^ used a two-layer guided aggregation layer to aggregate cost volumes, GwcNet^33^ built cost volumes by group correlation, and improved 3D stacked hourglass networks, both of which achieved better disparity prediction results. Although disparity estimation is highly accurate, this type of network usually requires a large amount of computation, especially in high-resolution stereo matching.

Compared with 3D convolution, 2D convolution has the advantages of faster inference and fewer parameters, which is more prevalent among researchers^15,16,20,34,35^. Mayer et al. proposed DispNet, with an encoding-decoding structure of the neural network^15^. The encoder uses a Siamese network that extracts a single feature and uses the left and right image features to calculate the correlation amount. Pang et al. designed a two-stage cascaded CNN structure with multi-scale residual learning based on DispNet^34^. Based on the optical flow network RAFT^35^, Lahav et al. proposed a rectification network RAFT-Stereo with multi-level Gate Recurrent Units (GRU)^20^, and Li et al. presented a three-layer cascaded recurrent network CREStereo with adaptive correlation^16^. In particular, CREStereo has been the first to introduce Transformer into the stereo matching task and attained state-of-the-art disparity estimation accuracy on the Middlebury^18^ and ETH3D^36^ datasets. CREStereo only considers the predicted disparity update impact of low-resolution on high-resolution features, however, the predicted disparity update impact of high-resolution on low-resolution features is ignored. Therefore, we design a multi-level network that considers the interaction between high and low-resolution features and adopts different group correlation layer strategies to update the disparity prediction values of different resolutions. The network can maintain a better accuracy of the disparity estimation while dramatically reducing the amount of model computation.

Besides the efficient model component of using 2D convolution instead of 3D convolution, many efficient model components^20,28,29,33,37,38^ have been proposed by various networks. A two-stage refinement network^29^ for stereo was first proposed by Liang et al.. Abhishek et al. designed Bi3D^28^, a depth estimation framework with a series of binary classifications, which could detect objects closer to a given distance within a few milliseconds. Yang et al. employed a hierarchical network from coarse to fine for efficient model inference^37^, but the disparity prediction accuracy was worse. Vladimir et al. presented HITNet^38^, a neural network structure for real-time stereo matching. Multi-resolution fast initialization steps are used for this network. It allows multi-layer feature information to be propagated at different levels but requires different model architectures to train on other data. Guo et al. used the group correlation layer instead of the full correlation layer^33^, which can drastically decrease the model computational effort. Lahav et al. proposed a slow-fast GRU that used fast GRU for real-time inference^20^, but fast GRU and slow GRU needed to be trained at least twice.

In this paper, we introduce a pair of efficient model components with group correlation to significantly reduce the computational effort of the model. We also design a pair of stacked inference structures with slow-fast, which can be trained only once for both versions of slow-fast disparity prediction.

## Methods

The overall network framework will be first introduced in this section Next, we present two key modules affecting the LMCR network: a pair of feature extractors and group correlation layers. Then, two more critical parts of the network are detailed: the LMCR network and the inference structure of the slow-fast stacked multi-level cascade. Followed by the loss function.

### Network architecture

**Figure 4:**
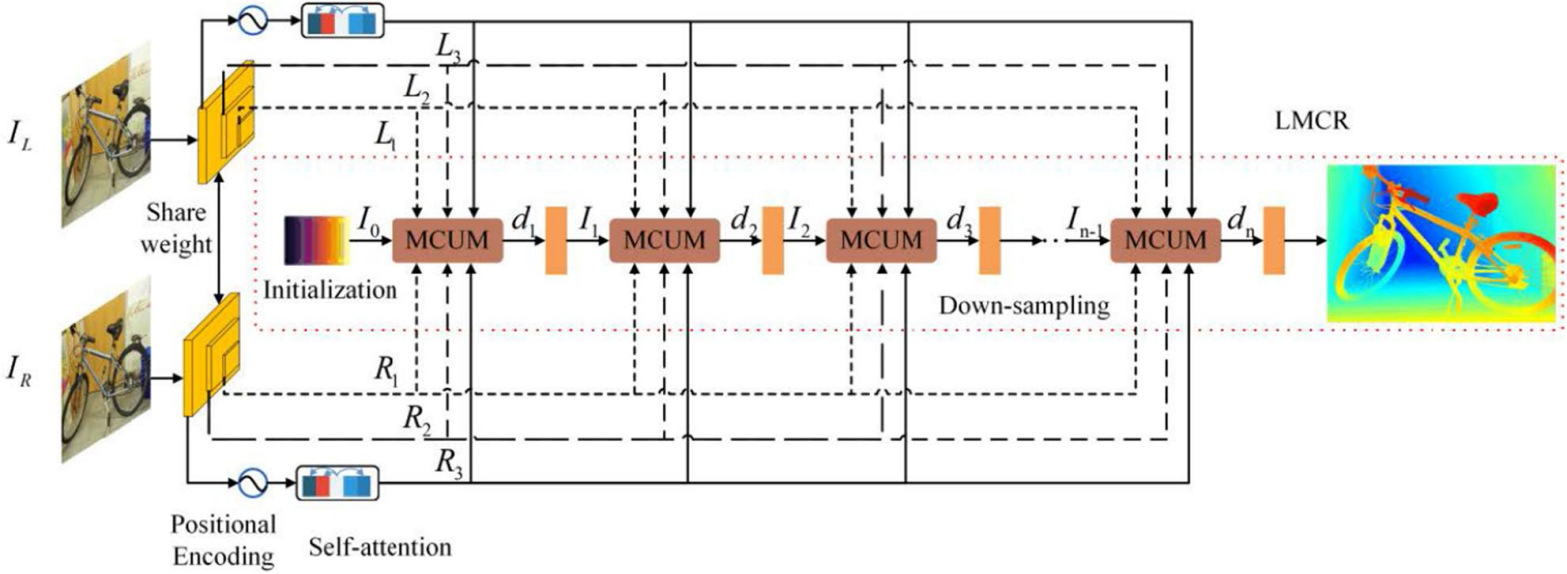
Overview of the network architecture. The network can be divided into LMCR, feature extractors, and group correlation layers.

The key to the LMCR-Stereo network framework is the LMCR network, as shown in Fig. 4. Moreover, two key modules that affect the prediction accuracy and speed of the LMCR network model include the feature extractors and the group correlation layers. Given a pair of calibrated stereo images ($I_{L}$, $I_{R}$), we first generate a three-layer feature pyramid network that is used to compute the group correlation of this network at different scales. The feature pyramid of $I_{L}$ also supplies contextual data for the recurrent update modules and offsets. Then, after outputting features at a higher feature pyramid resolution, the added positional encoding and self-attention provide global contextual data for the subsequent adaptive group correlation layer (AGCL). In addition, features and predictions are refined during the recurrent refinement phase using the multi-level cascaded update module (MCUM). The disparity predictions from the previous stage are down-sampled, with disparity initialization, and are used as the input values for the next recurrent step. For each iteration of MCUM, a pair of group correlation layers is used to calculate its correlation. Finally, we propose a dual version of slow-fast stacked multi-level cascaded architecture in the inference stage, which can better utilize multi-level contextual information and adapt to different dataset differences.

### Feature extractors

The feature extraction network we used is similar to the RAFT-stereo^20^, which includes the feature encoder and the context encoder. The network comprises a series of down-sampling layers and residual blocks for generating a 256-channel feature map, which resolution is 1/8 of the input image. It then receives feature maps with image resolutions of 1/16 and 1/32 by average pooling. The feature encoder structures the feature maps associated with the left and right images using instance normalization^39^ and the same weight.

The context encoder has the same framework as the feature encoder. Different from the instance normalization used by CREStereo^16^, which uses batch normalization^40^ to replace the instance normalization and is only applied to the left image. Contextual features for initializing the hidden states of the AGCL and the lightweight group correlation layer (LGCL) are used and injected into the GRU^35^ in each iteration of the AGCL and the LGCL.

### Group correlation layers

**Figure 5:**
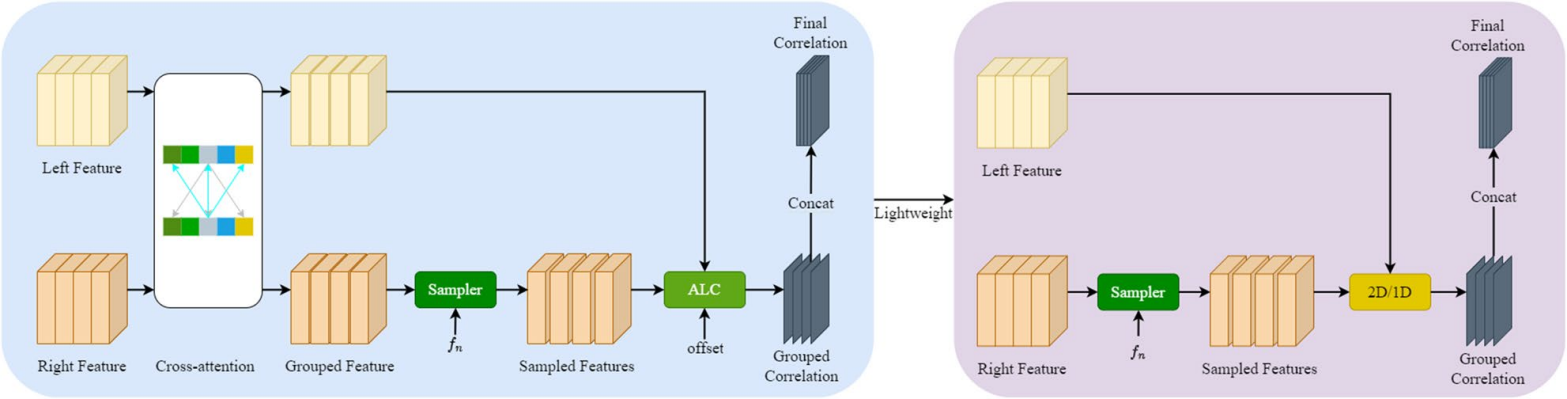
The proposed framework of group correlation layers. Left: AGCL, where ALC represents the adaptive local correlation; Right: LGCL.

To reduce the matching ambiguity because of imperfect rectification, CREStereo^16^ adopted an AGCL. AGCL requires great amounts of computation, however, increases the inference time dramatically. We need to improve the AGCL with a lightweight component. To speed up inference while ensuring accuracy as much as possible, we use a pair of group correlation layers, including the AGCL and the LGCL, as shown in Fig. 5. Two group correlation layer strategies are used for the iterative update of the model, i.e. LGCL in the two-layer feature maps with lower feature pyramid resolution and AGCL in feature maps with higher feature pyramid resolution. In the ablation experiment, we will discuss the performance of different combinations.

#### Local features attention

After the output of the higher-resolution feature map of the feature pyramid, the positional encoding is added and injected into an attention module^41^ for aggregating global contextual information into a single feature map or cross-feature map. This local feature attention is mainly used in the AGCL module and higher-resolution feature maps. Attention mechanisms have also been the subject of many recent studies^42^, and a number of these are also applicable to new approaches to image processing. The use of more advanced mechanisms may indeed enhance the computational speed of the present algorithm, but this study focuses on multi-layer networks, so we retain the original attention mechanism algorithm.

#### 2D-1D alternate local search

In traditional stereo matching tasks, the search direction between two calibrated images lies only on the epipolar line. To handle the case of non-ideal rectification, Li et al. proposed a 2D-1D Alternate Local Search strategy^16^. This method can save memory consumption and model computation significantly. In this paper, this strategy is applied to the LGCL module to enhance the inference speed of the model.

#### Lightweight group correlation layer

As shown in Fig. 5, we propose an LGCL module to speed up the inference of the model. This module removes the highly computational cross-attention and the corresponding offset component from AGCL and replaces ALC with a lightweight 2D-1D strategy. This strategy adopted by the improved module can significantly reduce the computational effort of this component.

### Lightweight-based multi-level cascaded recurrent network

It is more robust for matching regions with textureless or repeated textures by using low-resolution and high-level feature maps. That is the reason for having wide receptive fields and enough semantic information. However, fine structure details may be lost. To improve this situation, although the CREStereo network proposed by Li et al. can achieve good disparity estimation^16^, the model takes a long time to infer the predicted disparity. For this reason, we offer an LMCR network to update the disparity for each iteration.

#### Disparity initialization

By default, the disparity field of the image in 1/32 resolution is initialized to 0 everywhere. After experiments, we found that specific initialization of the initial disparity value leads to faster and better prediction of the best disparity. When performing disparity prediction, we define the initial disparity as:

1 $$\begin{aligned} P_{0} = \begin{bmatrix} 1&{} 2&{} \cdots &{} w&{} \\ \vdots &{} \vdots &{} \ddots &{} \vdots &{} \\ 2&{} 2&{} \cdots &{} w&{}\end{bmatrix} \end{aligned}$$

The predicted disparity $f_{pred}$ as:

2 $$\begin{aligned} f_{pred} = P - P_{0} \end{aligned}$$

where *P* denotes the predicted disparity in the middle of the model, $P_{0}$ denotes the initial disparity, $f_{pred}$ denotes the predicted disparity, and *w* denotes the width of the current feature pyramid.

#### Multi-level cascaded update module

In the CREStereo^16^ network, the Recurrent Update Module (RUM) is constructed based on GRU and AGCL. This module calculates the correlation of each feature map separately in different cascades, using $f_{0}$ as the initial input in each cascade and refining the differences of several iterations independently.

**Figure 6:**
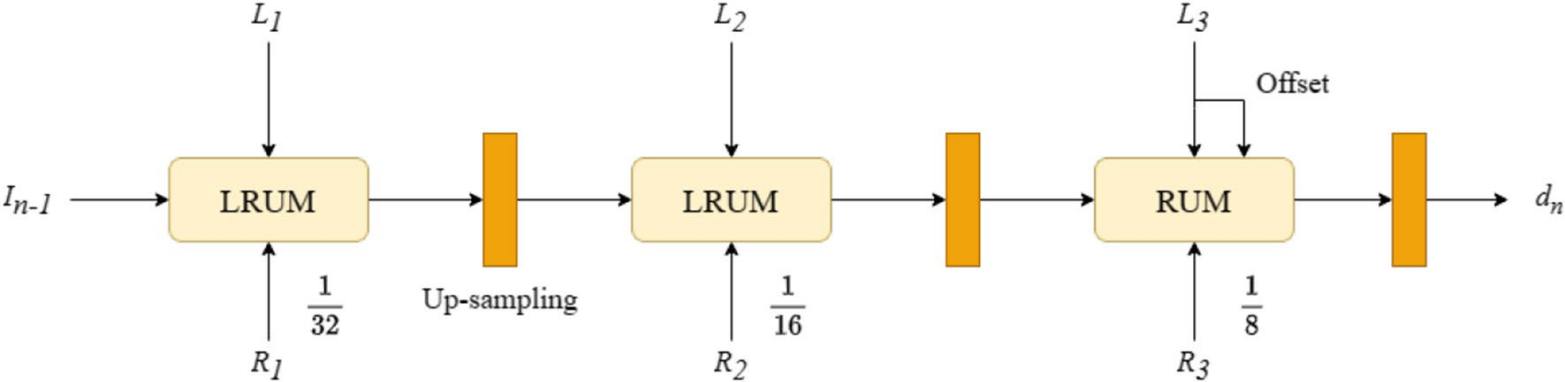
The proposed framework of the MCUM. That consists of a three-level feature pyramid that predicts the variance values through hierarchical recurrent refinement and cascaded refinement iterations. The module uses two update strategies, the LRUM, and the RUM.

Based on the lightweight design, we constructed the MCUM module. Figure 6 shows the module’s structure. It includes a RUM module, two Lightweight Recurrent Update Modules (LRUM), the n^th^ iteration input value $I_{n-1}$, and the left and right feature map input values $L_{i}$ and $R_{i}$. Where the RUM uses the same structure as CREStereo^16^, the LRUM is a replacement of the AGCL in the RUM with the LGCL. $L_{i}$ and $R_{i}$ are 1/32 and 1/16 of the original resolution in the left and right feature map input values. We adopt the LRUM to refine the difference values after iterations and update the corresponding disparity values. RUM is used to refine the differences after iterations in the feature map where the original image resolution is 1/8.

#### Iterative refinement strategies

We design three iterative refinement strategies in the model, namely hierarchical recurrent refinement, cascaded refinement, and recurrent refinement. The hierarchical recurrent refinement strategy includes three feature pyramids at 1/32, 1/16, and 1/8 of the model input resolution, with independent $n_{1}$, $n_{2}$, and $n_{3}$ iterations of refinement differences. For cascaded refinement strategy, except for the first cascade where the initialized disparity is equal to $P_{0}$, the other levels up-sample the predicted disparity value of the previous level plus the initialized disparity $P_{0}$ of that level as the initialized disparity value for that level of cascaded refinement. The recurrent refinement strategy means that except for the first cycle when the disparity value is initialized to $P_{0}$, the n^th^ cycle is to down-sample the disparity value $d_{n-1}$ output from the previous cycle plus $P_{0}$ as the initialized disparity value, for a total $n_{4}$ iterations to refine the disparity difference. Noteworthy, the process of one recurrent refinement must go through three hierarchical recurrent refinements and two cascade refinements. In this method, despite using different levels of hierarchical recurrent refinement, cascaded refinement, and other numbers of recurrent refinements, all parameters of the same part of the RUM and the LRUM share the same weights. After each recurrent refinement, an 8-fold convex up-sampling^35^ is performed, resulting in a disparity prediction at the input image resolution.

### Slow-fast stacked multi-level cascades for inference

**Figure 7:**
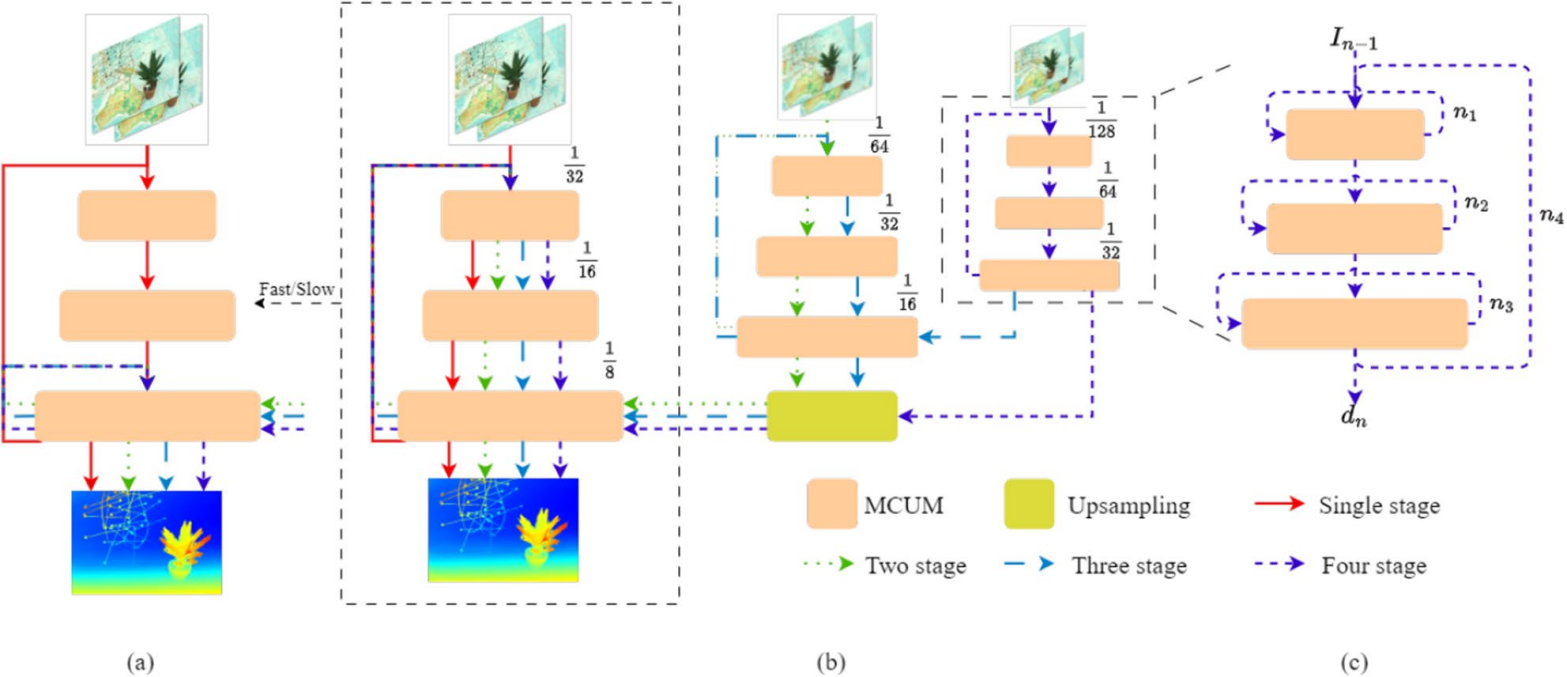
Our proposed slow-fast stacked cascaded architecture for inference. (**a**) A simplified diagram of the inference structure of the fast version. (**b**) A multi-level stacked inference structure of the slow version. (**c**) The recurrent routes between feature pyramids are omitted in (**b**).

As mentioned in previous sections, we employ a three-level feature pyramid for hierarchical recurrent refinement, cascaded refinement, and recurrent refinement. Using the high-resolution image as input, however, expands the receptive domain for extracting features and correlation calculations with the increase of the down-sampling factor. It may degrade the fine object features with the large displacement of that image simultaneously. We design a stacked multi-level cascaded inference structure to solve this problem. We down-sample the stereo image pairs in advance to construct a three-layer pyramid, which is then fed to the feature extractor at the same resolution used for training. Figure 7b and c show the stacked multi-level cascaded structure graph, and Fig. 7b does not show the hierarchical recurrent refinement and skip connections at the same stage for the sake of brevity. The inference structure offers two routes with different inference multiples of 2 or 4. The stacked multi-level cascaded structure shares the same weights in all phases during inference and training, so no fine-tuning is required.

In addition, this network employs the LGCL on the lower-resolution two-layer feature map to update the iterative disparity values. The disparity estimation accuracy of the AGCL in this network is better than that of the LGCL. When the input model size is larger than the original image size, the LGCL corrupts the accuracy of the final disparity prediction. For that, we also propose a Fast version of the stacked multi-level cascade structure, which constitutes a slow-fast dual version of the multi-level cascade stacked inference structure, as shown in Fig. 7a and b. For the Fast version, except for the initial time when the whole multi-level cascaded structure is used (as in Fig. 7c), only the higher resolution layer of the feature pyramid is used for disparity prediction. This version of the inference structure accelerates the model to predict disparity and reduces the prediction error caused by the LGCL at a higher resolution.

### Loss function

The excellent performance of CREStereo has demonstrated the superiority of the loss function it uses, and we have chosen to retain its methodology. Since this paper focuses on the design of the network, we have not modified the original loss function significantly. We added averaging to the original loss function to improve the stability of the model across batches and datasets, as well as reducing overfitting in small batches of training. For each stage $s \in (\frac{1}{32}, \frac{1}{16}, \frac{1}{8})$ in each cycle, the feature pyramid is adjusted according to the number of cycles $[n_{1}, n_{2}, n_{3}, n_{4}]$ in each stage of the output sequence $\{f_{1}^{s}, f_{2}^{s},\cdots , f_{i}^{s},\cdots , f_{n}^{s} \}$ as:

3 $$\begin{aligned} f = \{f_{1}^{s_{1}},\cdots ,f_{j_{1}}^{s_{1}} \mid f_{j_{1}+1}^{s_{2}},\cdots , f_{j_{2}}^{s_{2}} \mid f_{j_{3}}^{s_{3}},\cdots ,f_{j_{3}}^{s_{3}} \} \end{aligned}$$

Where $s_{1}$ denotes the resolution of 1/32 stage, $s_{2}$ denotes the resolution of 1/16 stage, and $s_{3}$ denotes the resolution of 1/8 stage, $j_{1} = n_{1}\times n_{4}, j_{2} = (n_{1} + n_{2})\times n_{4}, j_{3} = (n_{1}+n_{2}+n_{3})\times n_{4}$.

The predicted value $d_{s}$ under the input image resolution is obtained by up-sampling the output sequence *f* . The $l_{1}$ distance between the predicted value $d_{s}$ and the ground truth disparity $d_{gt}$ is supervised using exponential weighting, with exponentially increasing weights ($\lambda$ is set to 0.9). The total loss function is defined as:

4 $$\begin{aligned} L = \frac{1}{n} \sum _{i=1}^{n} {\textstyle \sum _{s}^{}\gamma ^{n-i} \Vert d_{gt} -d_{s}(f_{i}^{s}) \Vert _{1}} \end{aligned}$$

## Experiments

### Datasets

We trained and evaluated our approach on four stereo datasets: Middlebury 2014^18^, ETH3D^36^, SceneFlow^15^, and CREStereo dataset^16^.

Middlebury 2014 supplies 33 high-resolution image pairs of static indoor scenes under different lighting environments, captured with a large baseline stereo camera with up to 6 million pixels and a maximum disparity of more than 600. On this dataset, mainly AvgErr and Bad 2.0 metrics^2,36^ are used for evaluation.

ETH3D consists of 27 monochrome stereo images that were sampled by a laser scanner and covers both outdoor and indoor scenes. This dataset is mainly evaluated with AvgErr and Bad 1.0 metrics.

SceneFlow is a manually synthesized binocular stereo matching dataset using a virtual engine, which consists of two versions, “finalpass” and “cleanpass”, each with 35,454 training image pairs and 4,370 test image pairs. The stereo image pairs have a dense standard disparity map with a resolution of 540$\times$960. AvgErr and Bad 1.0 metrics are mainly used for evaluation on this dataset.

CREStereo provides a large synthetic dataset created manually, which has nearly 200,000 stereo image pairs in the training set. We randomly selected a total of 35,000 stereo image pairs from the four parts “hole”, “reflective”, “shapenet” and “tree” to make a simple CREStereo training set.

### Training schedule

Our network is implemented using the Pytorch^43^ framework and optimized using AdamW^44^ optimizer. The final model is trained on 1 NVIDIA GTX 3090Ti GPU, with a batch size of 4 for a total of 300,000 training iterations. The ablation experiments are trained using a batch size of 4, except for the inference structure ablation experiments with stacked multi-level cascades, which are trained in 40,000 iterations. We use a single-cycle learning rate schedule^45^ with a maximum learning rate of $2e^{-4}$. The size of all training inputs in the LMCR-Stereo network is 384$\times$512, and all training samples are augmented with a set of data before training. Specifically, asymmetric chromaticity enhancement, including luminance, contrast, and gamma shift, is applied to the left and right pair of input images. To avoid mismatching caused by unsuitable regions in natural scenes and to enhance robustness to rectification errors, random masked rectangular blocks between 50 and 100 pixels are used in the height and width directions. Also, random transformations and vertical offsets are applied only for a 2-pixel range of the right image. Finally, random resizing and cropping operations are performed on the stereo image pairs and disparity groups.

### Ablation experiments

In this section, we evaluate the LMCR-Stereo network under different settings to demonstrate the effectiveness of the settings of each network component. The evaluation resolution is 540$\times$960 for all except for the ablation study of the stacked multi-level cascade.

#### Iteration types

Li et al. have demonstrated that using 2D-1D alternative components is more conducive to achieving good accuracy when the RUM is used^16^. However, the work does not explore what ratio of hierarchical recurrent refinement and recurrent refinement iterations is taken to improve disparity prediction results. We use $n_{i}$ (where $i=[1, 2, 3]$) and $n_{4}$ to form a 2D-1D alternative search. With the total number of iterations roughly constant, the impact of different types of correlations is explored by varying the number of iterations and the correlation ratio of $n_{i}$ (where $i=[1, 2, 3]$). We adjust the number of iterations of recurrent refinement by increasing the proportion of the RUM or the LRUM in the overall number of iterations. As shown in type 1 of Table 4, when 2D or 1D search is used for $n_{1}$, $n_{2}$, and $n_{3}$, it corrupts the prediction accuracy of the model. When the alternating 2D-1D search is used, increasing the number of iterations $n_{4}$ of multi-level refinement can predict the disparity values in a better way. That is also proved by the results of other predictions in Table 4. In addition, when increasing the cycle refinement ratio of AGCL, the final accuracy of the model is lost, and the inference time of the model is increased due to reducing the number of recurrent refinements $n_{4}$. Based on the consideration of lightweight, we adopt the iteration combinations from type 3 to type 6 as shown in Table 4. It increases the proportion of LGCL, accelerates the model inference, and achieves a good balance between higher accuracy and faster speed at type 6.

**Table 4:** Ablation study for iteration types.

Modle		$n_{1}$			$n_{2}$			$n_{3}$			AvgErr	Bad 1.0	Time (s)
		1D	2D	2D/1D	1D	2D	2D/1D	1D	2D	2D/1D			
Type 1	[1,1,1,12]	$\checkmark$			$\checkmark$			$\checkmark$			**1.201**	13.72	0.558
	[1,1,1,12]		$\checkmark$			$\checkmark$			$\checkmark$		1.354	13.72	0.449
	[2,2,2,6]			$\checkmark$			$\checkmark$			$\checkmark$	1.269	13.17	0.441
	[3,3,3,4]			$\checkmark$			$\checkmark$			$\checkmark$	1.223	13.40	0.475
Type 2	[1,1,2,9]	$\checkmark$			$\checkmark$				$\checkmark$		1.346	13.53	0.552
	[3,3,6,3]			$\checkmark$			$\checkmark$			$\checkmark$	1.273	14.44	0.443
Type 3	[2,1,1,9]			$\checkmark$	$\checkmark$			$\checkmark$			1.252	13.34	0.445
	[4,2,2,4]			$\checkmark$			$\checkmark$			$\checkmark$	1.231	**12.98**	0.410
Type 4	[3,2,1,6]			$\checkmark$			$\checkmark$	$\checkmark$			1.266	13.03	0.358
	[6,4,2,3]			$\checkmark$			$\checkmark$			$\checkmark$	1.234	13.08	0.370
Type 5	[4,3,2,4]			$\checkmark$			$\checkmark$			$\checkmark$	1.267	13.08	0.397
Type 6	[5,3,1,4]			$\checkmark$			$\checkmark$	$\checkmark$			1.267	13.03	**0.333**

The AvgErr and Bad 1.0 metrics are measured on SceneFlow test set.

Significant values are in bold.

#### Components in MCUM

As shown in Table 5, we compare the behavior of different AGCL and LGGL with different ratios and locations. The AGCL can effectively suppress the error of prediction disparity. When the combination of $1 AGCL + 2 LGCL$ is used, and the AGCL is at the higher resolution position of the feature pyramid, the model achieves the best speed and prediction effect. In addition, we verify that the four-level refinement predicts disparity values more accurately than the traditional three-level refinement. In the same experimental setting, compared with the prediction effect and inference speed of CREStereo^16^, the model inference time is reduced by 52%, the inference speed is faster, and the prediction effect is better. It demonstrates the effectiveness of our lightweight mode mechanism.

**Table 5:** Ablation study for the MCUM.

Model	AvgErr	Bad 1.0	Bad 3.0	Time (s)
CREStereo^16^, 3 levels	1.434	14.90	6.31	0.695
2 AGCL + 1 LGCL, 3 levels	1.284	15.24	5.72	0.372
2 AGCL + 1 LGCL, 4 levels	**1.243**	14.08	5.49	0.381
1 AGCL + 2 LGCL, 1/32, 4 levels	1.548	18.59	7.46	0.479
1 AGCL + 2 LGCL, 1/16, 4 levels	1.339	14.33	5.78	0.392
1 AGCL + 2 LGCL, 1/8, 3 levels	1.265	13.94	5.44	0.342
1 AGCL + 2 LGCL, 1/8, 4 levels (Ours)	1.252	**12.88**	**5.25**	**0.335**
No initialization	1.738	26.16	8.53	0.349
Instance normalization	1.334	13.89	5.52	0.433

The AvgErr, Bad 1.0, and Bad 3.0 metrics are measured on SceneFlow test set.

Significant values are in bold.

#### Features for refinement

**Figure 8:**
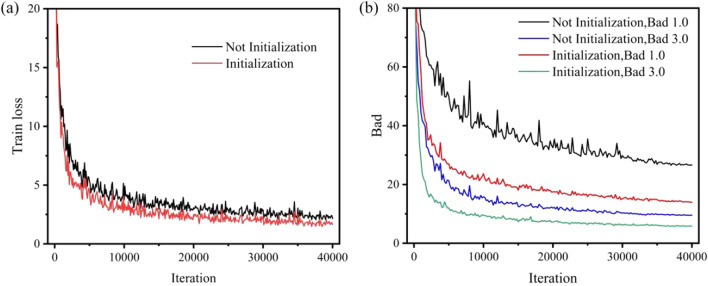
The effect of initializing disparity to $P_{0}$. (**a**) Improvement in training loss. (**b**) Improvement in Bad 1.0 and Bad 3.0.

From Table 5, the network without initialization reduces the speed of loss drop during model training and the final disparity prediction accuracy. Figure 8 shows the trend without presetting the differential disparity values. In addition, we also use instance normalization in the context encoder and set the same parameters at the feature encoder and context encoder parameters, which reduces the model’s number of parameters but loses some accuracy.

#### Stacked multi-level cascades

We employ the image pyramids of different levels as input in the inference process and share common model training parameters to predict the final disparity value after a multi-stage, multi-level stacked inference structure. We compare the performance of stacked multi-layer cascades at different resolutions, as shown on the left of Table 6. When only one multi-layer cascade is used, the prediction error decreases sharply with an increasing model input size. When using multi-level cascaded inference, low-resolution features do not affect the parallax prediction values at high-resolution images. As the input resolution decreases, the accuracy of the predicted disparity does not change and only the inference speed changes, where the inference speed of the Four-stage is the fastest.

#### Slow-fast inference

**Figure 9:**
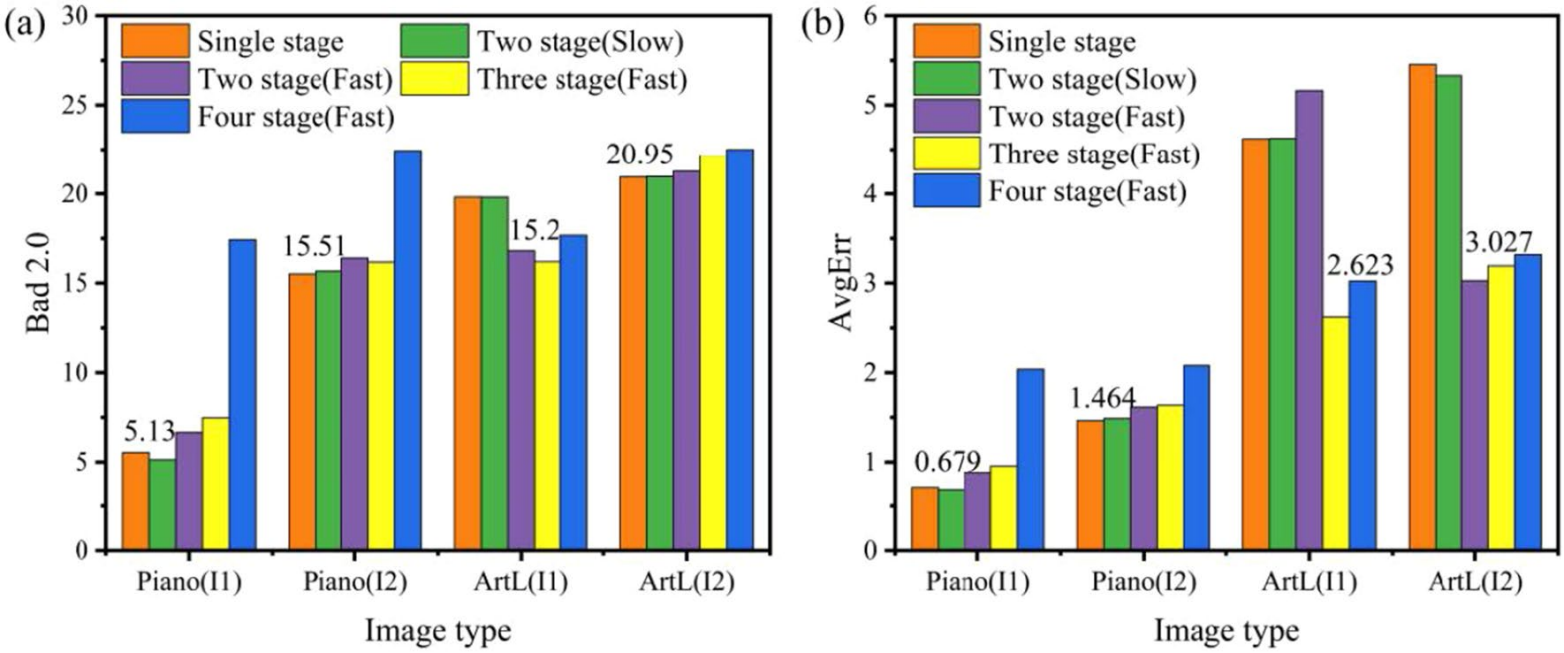
Ablation study of stacked multi-level cascaded architecture during inference on Piano and ArtL of Middlebury Datasets. The image size of ArtL is 1108$\times$1388, and the image size of Piano is 1924$\times$2828. I1 denotes the model input size of 1536$\times$2048, and I2 represents the model input size of 768$\times$1024. (**a**) Comparison of Bad 2.0 with different inference structures. (**b**) Comparison of AvgErr with different inference structures.

As shown in Table 6 and Fig. 9, we explore the prediction effects of the Slow-Fast stacked multi-level cascade structures when the relative relationship between the model input size and the predicted image size varies. Among them, the image prediction accuracy of ArtL in the high-resolution Middlebury dataset is inconsistent. Specifically, the slow version of the Two-stage stacked inference structure predicts better disparity accuracy when the model input size is smaller than the expected image size, as demonstrated by the result on Piano in Fig. 9. However, when the model input size is larger than the predicted image size, the fast version of the Three-stage stacked inference structure predicts disparity better and faster. Combined with Table 6, the inference results show that the multi-level stacked structure is beneficial in reducing the prediction error in both Middlebury and ETH3D datasets, but the best performance is different. Therefore, choosing the appropriate model input size and corresponding inference structure helps achieve a balance between accuracy and speed in predicting disparity, which demonstrates the effectiveness of our proposed slow-fast inference structure.

**Table 6:** Ablation study of stacked multi-level cascaded architecture during inference on Middlebury and ETH3D training set.

Method		Middlebury				ETH3D			
		Input size	AvgErr	Bad 2.0	Time (s)	Input size	AvgErr	Bad 1.0	Time (s)
Slow	Single stage	768$\times$1024	2.844	20.50	**0.465**	384$\times$512	0.228	2.18	0.508
	Single stage	1536$\times$2048	1.201	7.69	1.032	768$\times$1024	0.245	1.84	**0.449**
	Two stages	768$\times$1024	2.846	20.52	0.779	384$\times$512	0.229	2.29	0.753
	Two stages	1536$\times$2048	**1.196**	**7.61**	1.230	768$\times$1024	0.245	1.82	0.791
	Three stages	1536$\times$2048	**1.196**	**7.61**	1.575	768$\times$1024	0.245	1.82	1.126
	Four stages	1536$\times$2048	**1.196**	**7.61**	1.163	768$\times$1024	0.245	1.82	0.769
Fast	Two stages	768$\times$1024	3.338	22.17	0.637	384$\times$512	0.274	2.95	0.565
	Two stages	1536$\times$2048	1.646	10.41	1.131	768$\times$1024	**0.207**	**1.52**	0.634
	Three stages	1536$\times$2048	2.048	11.57	1.328	768$\times$1024	0.215	1.62	0.809
	Four stages	1536$\times$2048	2.966	15.18	1.079	768$\times$1024	0.236	2.03	0.622

Significant values are in bold.

#### Training

**Figure 10:**
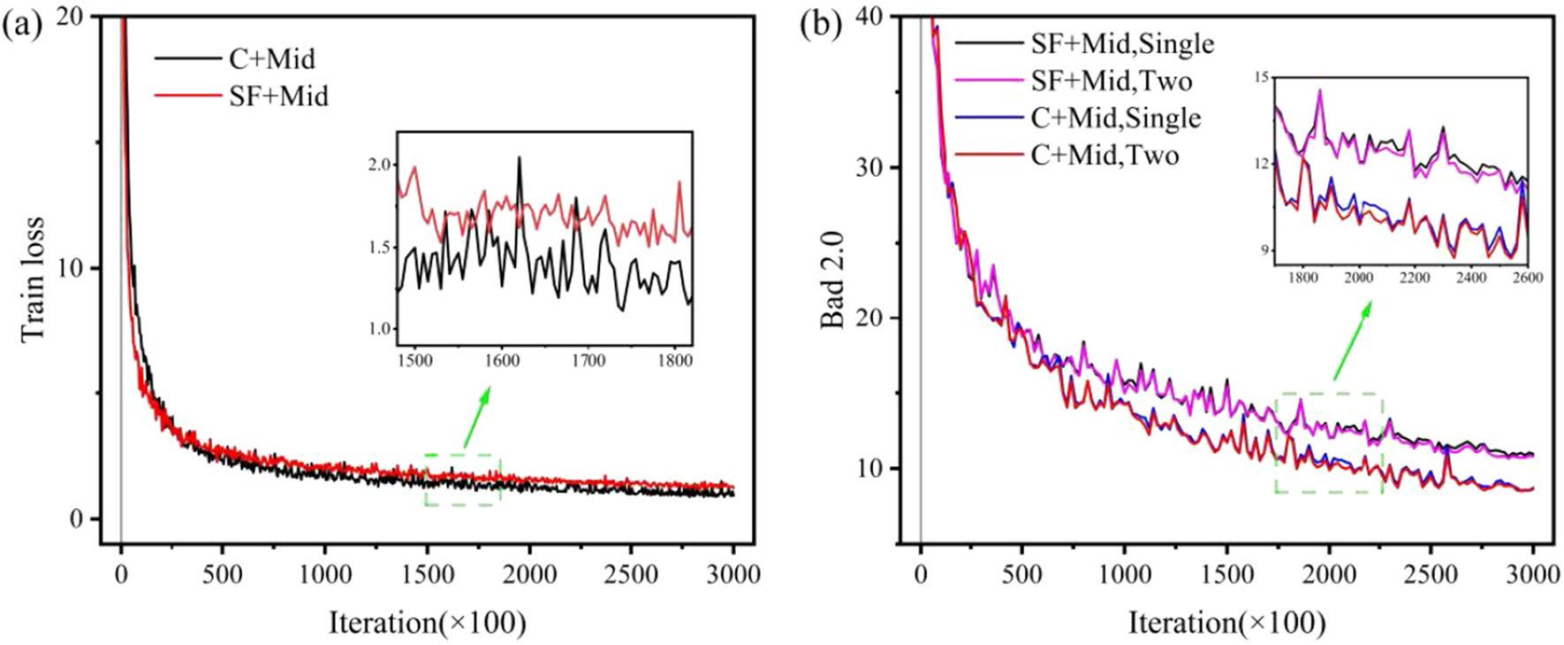
Training loss and Middlebury validation error of models trained with SceneFlow and CREStereo dataset, where C represents CREStereo dataset, SF represents SceneFlow dataset, and Mid represents Middlebury dataset. (**a**) Comparison of training loss with different datasets. (**b**) Comparison of Bad 2.0 with different inference structures.

We use different datasets and the Middlebury dataset combination for training and compare the loss convergence during training. Figure 10a shows CREStereo has better convergence and more accurate disparity prediction than Middlebury. Figure 10b highlights the fact that the inference structure of the Two-stage is more accurate than the inference structure of the Single-stage in disparity prediction.

## Data Availability

The data supporting the findings of this study are available within the paper. The associated pre-processed raw data is available and can be shared with interested parties upon reasonable request. Please contact the corresponding author for more information.

## Code availability

Our code is avaliable on https://github.com/bravester/LMCR-Stereo. Please contact the corresponding author for more information.
